# Multimodal kidney‐preserving approach in localised and locally advanced high‐risk upper tract urothelial carcinoma

**DOI:** 10.1002/bco2.113

**Published:** 2021-10-11

**Authors:** Omar Alhalabi, Matthew T. Campbell, Lianchun Xiao, Ana C. Adriazola, Nathaniel R. Wilson, Arlene O. Siefker‐Radtke, Paul G. Corn, Amado Zurita, Eric Jonasch, Jianjun Gao, Mehrad Adibi, Ashish M. Kamat, Neema Navai, Louis L. Pisters, Colin Dinney, Surena F. Matin, Amishi Y. Shah

**Affiliations:** ^1^ Department of Genitourinary Medical Oncology, Division of Cancer Medicine University of Texas MD Anderson Cancer Center Houston TX USA; ^2^ Department of Biostatistics, Division of Cancer Medicine University of Texas MD Anderson Cancer Center Houston TX USA; ^3^ Department of Internal Medicine University of Texas Health Science Center at Houston Houston TX USA; ^4^ Department of Urology, Division of Surgery University of Texas MD Anderson Cancer Center Houston TX USA

**Keywords:** Bacillus Calmette–Guerin, chemotherapy, endoscopic resection, immunotherapy, mitomycin, upper tract urothelial carcinoma

## Abstract

**Objectives:**

Multimodal kidney‐preserving (MKP) strategies may be an option for patients with localised or locally advanced high‐risk upper tract urothelial carcinoma (UTUC) who have a relative contraindication for nephroureterectomy (NU).

**Materials and methods:**

We studied patients with UTUC who were managed with MKP strategies, consisting of systemic anticancer therapy, with or without local/topical strategies after endoscopic control of intraluminal tumours. Primary end points were overall survival (OS) and progression‐free survival (PFS).

**Results:**

Fourteen patients received MKP treatment between August 2013 and April 2020. Median baseline estimated glomerular filtration rate was 43 mL/min/1.73m^2^. MKP was mainly pursued to avoid dialysis (10/14, 71%), followed by low performance status and/or comorbidities (2/14, 14%). All patients had received systemic therapy: chemotherapy (64%) and immunotherapy (36%). Endoscopic control and/or laser ablation was feasible in 7 (50%) patients. Calculated overall risk of non‐organ confined disease was 35%. Predicted 2‐year and 5‐year relapse‐free probability (RFP) was 74% (24–92%) and 62% (10–85%), respectively. Median follow‐up was 31 months (95% CI: 22.6, NE), median OS was 48.1 months (95% CI: 48.1, NE) and 2‐year OS probability was 0.89 (95% CI: 0.71, 1). Median metastases‐free survival was 48.1 months (95% CI: 26.8, NE), median PFS was 22.4 months (95% CI: 15.6, NE) and 2‐year PFS probability was 0.48 (0.26, 0.89).

**Conclusion:**

Management of high‐risk localised or locally advanced UTUC with MKP strategies was associated with good tolerance, preservation of renal function, and comparable PFS and OS to predicted in vulnerable patients. Prospective studies with more patients are needed to evaluate these possible benefits relative to current standards.

## 1 INTRODUCTION

Nephroureterectomy (NU) is part of the standard‐of‐care treatment for high‐grade or clinically infiltrating upper tract urothelial carcinoma (UTUC) and includes the removal of the entire kidney, ureter and ipsilateral bladder cuff.^1^, ^2^ An estimated 60% of UTUC have a muscle invasive tumour (i.e., ≥T2) at time of diagnosis as compared with 15–25% of bladder tumours, and understaging of UTUC on imaging is frequent.^3^, ^4^ Invasive tumours significantly impact outcomes in UTUC with a 5‐year cancer‐specific‐mortality free rate of 50–60%, as compared with 80–90% in non‐invasive tumours.^5^, ^6^

Recent data have highlighted the benefit of perioperative systemic therapy for high‐risk UTUC, and surgery has long remained an important consolidative strategy in this disease.^7^, ^8^, ^9^, ^10^ However, there are indications for multimodality kidney‐preserving (MKP) approaches, which may be alternatives to NU in select patients where surgery may carry higher morbidity, such as patients with bilateral involvement, tumours in a solitary kidney, chronic kidney disease (CKD) or poor performance status. This is a relatively common scenario that can arise for patients with underlying CKD, which is strongly associated with UTUC,^11^ and who have multiple synchronous or metasynchronous recurrences of UTUC. Current MKP modalities are indicated largely for low‐grade tumours and may be delivered surgically or endoscopically, through either antegrade or retrograde access, and are well described in the literature.^1^, ^2^ Endoscopic management of UTUC is associated with high recurrence rates, which can be reduced by application of topical therapy such as mitomycin.^12^, ^13^ However, there is minimal data for outcomes in UTUC patients who have a relative contraindication for NU and higher risk disease associated with higher rates of progression. In these cases, a ‘non‐traditional’ approach using endoscopic local control in addition to systemic management to manage an invasive component may provide an alternative to NU and dialysis or severe CKD.

The objective of the present study is to evaluate the clinical characteristics and treatment outcomes of patients with localised or locally advanced high‐risk UTUC with vulnerable kidney function or who refused standard of care options, focusing on the ability of this strategy to prevent NU or dialysis and progression to metastasis.

## 2 MATERIALS AND METHODS

### 2.1 Patients and follow‐up protocol

Baseline characteristics and clinical outcomes were retrospectively collected for patients with localised or locally advanced high‐risk UTUC who were treated at the University of Texas MD Anderson Cancer Center (MDACC), Houston, Texas. All patients had baseline and surveillance chest, abdomen and pelvis imaging with contrast enhanced computed tomography or magnetic resonance imaging with or without gadolinium depending on contraindications, as well as cystoscopy with ureteroscopy. Patients were followed with serial imaging every 3 months in the first 1–2 years then every 6 months for at least 2 additional years; urine cytology was not performed a as part of routine surveillance but may have been obtained selectively. Ureteroscopy was done for biopsy, disease assessment and, if feasible, attempted local control using a Holmium or diode laser. Patients did not undergo serial ureteroscopy. In cases of apparent radiographic complete response (CR) or near CR, a ‘confirmatory look’ ureteroscopy with laser ablation of any residual disease was performed. Patients with locally advanced or node positive (N2+) disease were excluded. This study was approved by the Institutional Review Board (IRB) of MDACC protocol RCR05‐0521 with waiver of informed consent. In order to provide a reference, we used the preoperative nomogram reported by Petros et al. to predict risk of non‐organ confined (pT3–4 or pN+) disease^14^ and the nomogram reported by Freifeld et al. to predict disease recurrence following radical NU.^15^

### 2.2 End points

End points of interest included overall survival (OS), 5‐year OS probability, progression‐free survival (PFS) and 5‐year dialysis‐free probability. Given that many patients had history of concordant or discordant tumours elsewhere in urothelial tract, date of diagnosis was defined as that for the UTUC managed with ‘non‐standard’/MKP approach. OS was calculated as the duration from diagnosis to date of death or to the date of last follow‐up for patients alive. Two‐year and 5‐year OS probability was calculated as the percentage of patients alive at 2 and 5 years from diagnosis, respectively. PFS was calculated as the duration from diagnosis to date of progression (local and distant) or date of death, whichever occurred first, or to the date of last follow‐up for patients alive and without progression. Five‐year dialysis‐free probability was calculated as the percentage of patients free from dialysis at 5 years from diagnosis.

### 2.3 Statistical analysis

Continuous variables were summarised using descriptive statistics, and categorical variables were tabulated with frequency and percentage. The Kaplan–Meier method was used to estimate the time to event outcomes, and the log rank test was used to compare these outcomes between subgroups of patients. SAS software v9.4 (SAS Institute Inc., Cary, NC) and Splus software v8.2 (TIBCO software Inc., Palo Alto, CA) were used for statistical analysis.

## 3 RESULTS

### 3.1 Baseline characteristics of patients

Between August 2013 and April 2020, of 353 patients with UTUC, 21 were recommended treatment using MKP strategies, and 7 did not return to our centre for treatment, leaving 14 who are the subject of this study. Table 1 summarises the baseline clinical characteristics of the patients. Median age was 74 (range: 57–89), 50% (7/14) of patients were male and 43% had a history of smoking. Median estimated glomerular filtration rate (eGFR) at baseline was 43 mL/min/1.73m^2^ (range: 22–87). Avoiding dialysis was the most common reason (10/14, 71%) to elect MKP strategy over NU; 43% (6/14) of patients had prior contralateral nephrectomy. Low Eastern Cooperative Oncology Group (ECOG) performance status (PS) of 2–3 was the second most common reason to avoid surgery, occurring in 14% (2/14). Two‐thirds of the patients had a history of a prior UTUC, and half had a prior bladder cancer; the prior management of these 10 patients with previous urothelial cancer diagnoses is included in Table 1. Table 2 summarises the baseline tumour‐related characteristics. The majority (13/14) of patients had pure urothelial carcinoma histology, and 93% (13/14) had a high‐grade biopsy. Deficient DNA mismatch repair (dMMR) was found in 28% (4/14) of patients. Most patients (12/14) had hydronephrosis at presentation or required ureteral stenting or percutaneous nephrostomy. The overall risk of non‐organ confined disease was 35% for all patients per the Petros multiplex nomogram. The predicted 5‐year relapse‐free probability (RFP) was 63% (range: 10–85) per the Freifeld predictive model.

**TABLE 1:** Patients' baseline clinical characteristics (*n* = 14)

	Variable	Frequency (%)
Age (median, range)	‐	74, 57–89
Sex	Male	7 (50%)
	Female	7 (50%)
Race	White	12 (86%)
	Hispanic	2 (14%)
ECOG PS	0	6 (43%)
	1	5 (36%)
	2	2 (14%)
	3	1 (7%)
Smoking history	Never	8 (57%)
	Prior or current	6 (43%)
Estimate smoking pack‐year (median, range)	‐	2.5–0, 59
History of bladder cancer	Ta	4 (28%)
	T1	3 (22%)
Prior therapy for bladder cancer	BCG	3 (22%)
	Cisplatin‐based neoadjuvant chemotherapy	1 (7%)
In relation to upper tract lesion	Synchronous^a^	1 (14.3%)
	Metachronous^a^	6 (85.7%)
History of a prior upper tract lesion	High grade	7 (50%)
	Low grade	2 (14%)
Prior therapy for upper tract cancer	Cisplatin‐based neoadjuvant chemotherapy	2 (14%)
In relation to upper tract lesion	Synchronous^a^	2 (14%)
	Metachronous^a^	7 (50%)
eGFR, mL/min/1.73 m^2^ (median, range)	Pretreatment	43, 22–87
	Post‐treatment^b^	43, 26–105
Hydronephrosis at baseline	No	2 (14%)
	Yes	12 (86%)
Reason to avoid surgery	Avoid dialysis	10 (71%)
	Low PS and comorbidities	2 (14%)
	Patient preference	2 (14%)
Prior surgeries	None	6 (43%)
	Nephrectomy/nephroureterectomy^c^	6 (43%)
	Ureterectomy	2 (13.3%)

^a^ Synchrony was defined as occurrence within 3 months of the upper tract lesion.

^b^ Post‐treatment eGFR was available for 10 patients who received systemic therapy.

^c^ One patient had prior cystectomy, in addition to nephroureterectomy.

Abbreviations: ECOG PS: Eastern cooperative oncology group performance status, eGFR: estimated glomerular filtration rate.

**TABLE 2:** Patients' baseline tumour‐related characteristics (*n* = 14)

Variable	Value	Range or frequency (%)
Tumour location	Renal pelvis	11 (79%)
	Ureter	2 (14%)
	Both	1 (7%)
Tumour laterality	Bilateral	1 (7%)
	Unilateral	13 (93%)
Sessile tumour architecture	No	7 (50%)
	Yes	2 (14%)
	Unknown	5 (36%)
Clinical nodal staging	cN0	10 (72%)
	cNx^a^	3 (21%)
	cN1	1 (7%)
Haemoglobin (mean ± SD)	‐	11.9 ± 2.3
Biopsy grade	High	13 (93%)
	Low	1 (7%)
Histology	Pure UC	13 (93%)
	UC with variant histology^b^	1 (7%)
Microsatellite instability	Low	8 (58%)
	High	4 (28%)
	Unknown	2 (14%)
Predicted probability of pT3–pT4 and/or N+ at surgery	Overall	35% (20, 89)
Predicted probability of relapse‐free survival (median, range)	2‐year	74% (24, 92)
	5‐year	63% (15, 85)

Abbreviation: UC, urothelial carcinoma.

^a^ cNx refers to patients with borderline clinical locoregional lymph node disease for sizes ranging between 0.8 and 1.3 cm.

^b^ One patient had squamous cell carcinoma features on histology.

### 3.2 Utilised MKP treatment strategies

Prechemotherapy endoscopic control and/or laser ablation was feasible in 7 (50%) patients. All patients received systemic therapy (Table 3), which included immunotherapy (36%), cisplatin‐based chemotherapy (29%), carboplatin‐based chemotherapy (14%) and non‐platinum‐based chemotherapy (21%). Median number of cycles of systemic therapy was 3 (range: 2–12). Eight (57%) patients required change in systemic therapy due to worsening renal function (12.5%), cytopenia (25%) and poor tolerance (62.5%). Following systemic therapy, five patients required subsequent local/topical control with laser ablation. The detailed management and outcomes for the 14 patients with high‐risk UTUC treated with MKP strategies are listed in Table S4.

**TABLE 3:** Multimodality kidney‐preserving treatment strategies

Variable	Value	Range or frequency (%)
Required ureteral stenting or PCN	No	2 (14%)
	Yes	12 (87%)
Prechemotherapy local/topical control strategy^a^	None	6 (43%)
	Endoscopic biopsy	4 (29%)
	Laser ablation	3 (21%)
	Gemcitabine	2 (13.3%)
Systemic therapy regimen	Pembrolizumab	4 (29%)
	GTA	2 (14%)
	CGA	1 (7%)
	ddMVAC	1 (7%)
	Gem/cis	2 (14%)
	Gem/carbo	2 (14%)
	Atezolizumab	1 (7%)
	IA‐Gem	1 (7%)
Number of cycles (median, range)	‐	3 (2, 12)
Subsequent local/topical control strategy^a^	None	9 (64%)
	Laser ablation	5 (36%)
	BCG	2 (14%)
	Gemcitabine	1 (7%)
Required change in systemic therapy	Yes	8 (57%)
Reason for change in systemic therapy	Worsening renal function	1 (12.5%)
	Cytopenias	2 (25%)
	Poor tolerance	5 (62.5%)

Abbreviations: CGI, cisplatin, gemcitabine and ifosfamide; dd‐MVAC, dose‐dense methotrexate, vinblastine, adriamycin and cisplatin; Gem/carbo, gemcitabine and carboplatin; Gem/cis, gemcitabine and cisplatin; GTA, gemcitabine, taxotere and adriamycin; IA‐Gem, ifosfamide, adriamycin and gemcitabine; PCN, percutaneous nephrostomy.

^a^ Five patients underwent multiple local/topical therapy modalities explaining why the total number in this group is above total cohort of 14.

### 3.3 Survival and organ‐preservation analysis

**FIGURE 1:**
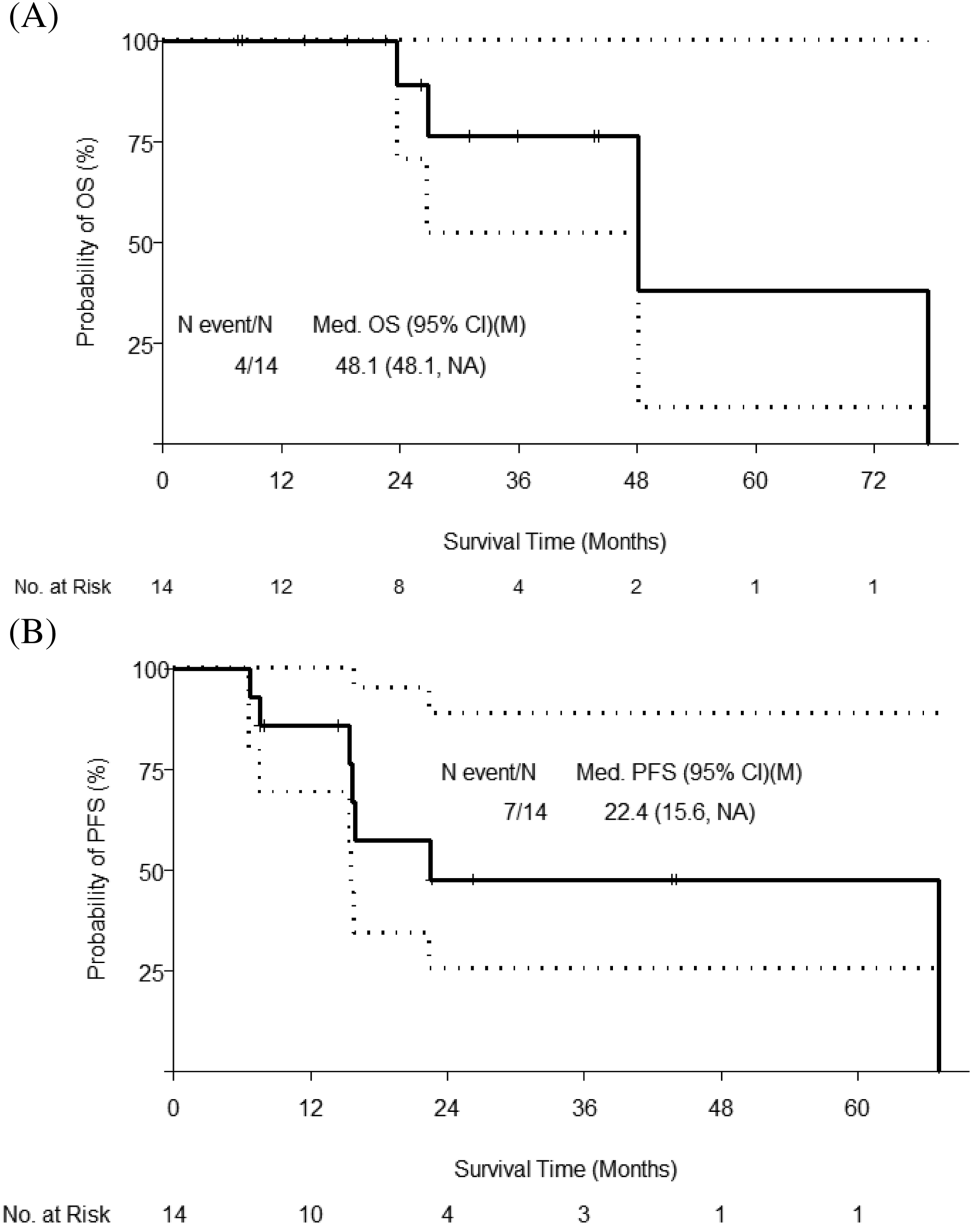
Overall and progression‐free survival

At a median follow‐up time of 31 months (95% CI: 22.6, NA), 4 of the 14 patients died, the estimated median OS was 48.1 months (95% CI: 48.1, NA) (Figure 1), 2‐year OS probability was 0.89 (95% CI: 0.71, 1) and 5‐year OS probability was 0.38 (95% CI: 0.09, 1) (Table S5). Additionally, 6 of the 14 patients developed distant metastases or died; the estimated median distant metastases‐free survival time was 48.1 months (95% CI: 26.8, NA). When considering both local and distant progression, the estimated median PFS was 22.4 months (95% CI: 15.6, NA), and the 2‐year PFS probability was 0.48 (0.26, 0.89). In terms of organ preservation, 3 of the 14 patients ultimately underwent NU as a result of local progression; the estimated 5‐year NU‐free probability was 0.6 (95% CI: 0.31, 1). Median posttreatment eGFR was 43 (range: 26–105). Furthermore, 2 of the 14 patients had progressive CKD and ultimately required haemodialysis, with an estimated 5‐year dialysis free rate of 0.75 (95% CI: 0.5, 1). Overall, 50% of patients achieved the ‘trifecta’ of no dialysis, NU or metastasis.

## 4 DISCUSSION

**FIGURE 2:**
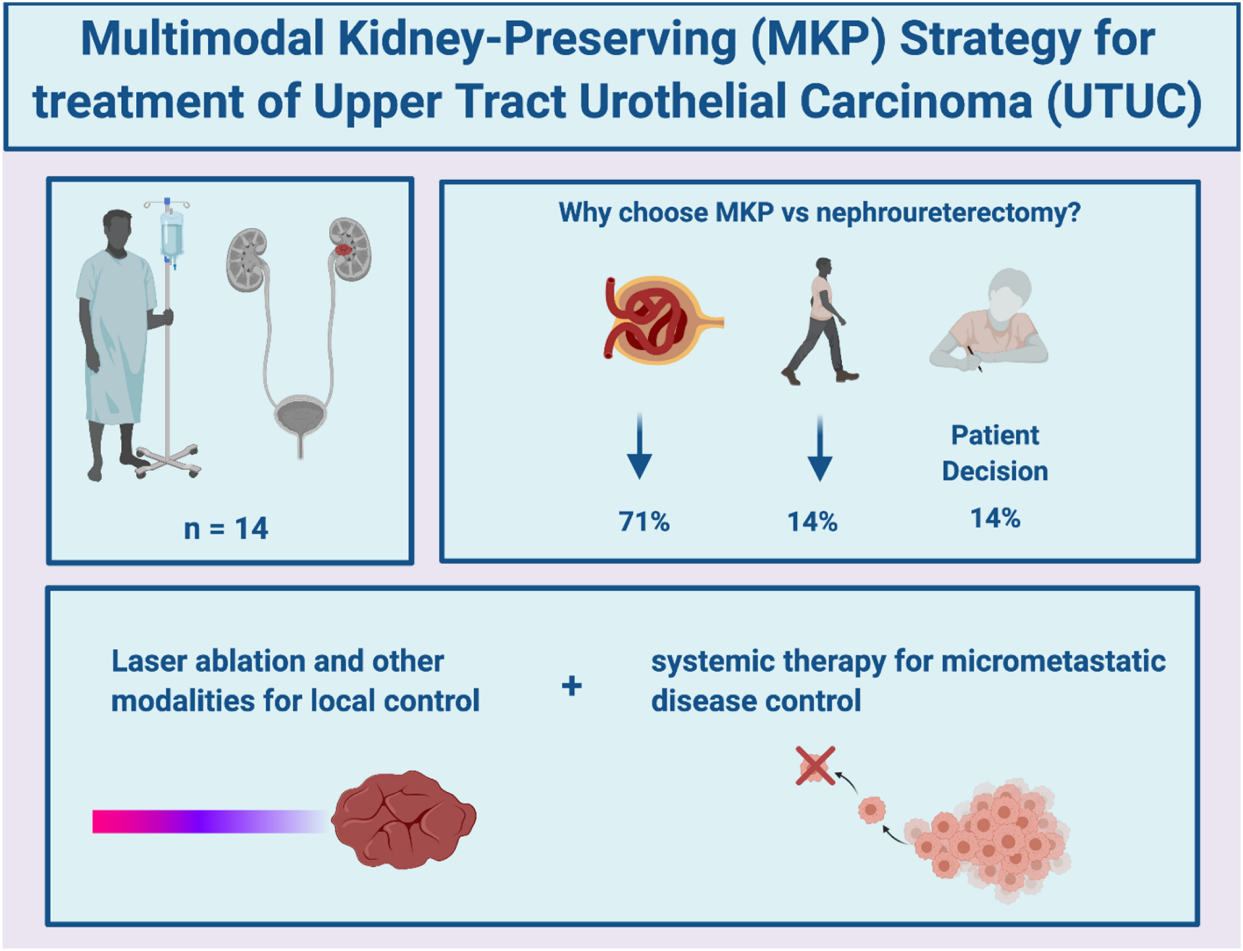
Rationale for multimodal kidney‐preserving strategy for treatment of upper tract urothelial carcinoma. Figure created using licenced version of biorender.com

We report on our experience using MKP management strategies to treat challenging and under‐recognised scenarios of localised or locally advanced high‐risk UTUC with relative contraindications for NU. Contraindications to NU were either due to poor baseline renal function and/or poor PS with surgery‐prohibitive comorbidities. The 5‐year OS probability rate was 38% (95% CI 9–100%) with MKP management strategies, which might initially appear lower than historic 5‐year OS rate of 80.2%^16^ as reported in a cohort of 31 patients who were able to receive neoadjuvant chemotherapy followed by NU. However, this group of patients has generally not been a focus of prior studies, is excluded in clinical trials and is largely ignored in clinical guidelines. The majority of patients in our analysis have unfavourable disease characteristics that have been correlated with inferior survival in UTUC, such as preoperative hydronephrosis, and reduced baseline renal function.^14^, ^15^ A more equivalent group may be patients with high‐grade disease who undergo endoscopic management. The review by Cutress^17^ and colleagues concluded that endoscopic management of high‐grade disease had poor outcomes and should only be considered for compelling imperative indications. The Cutress data showed a 5‐year disease‐specific survival of 60–79% but pointed out that this is likely an overestimate due to biassed reporting and raw figures uncensored to overall vital status; in fact, studies that appropriately censored patients showed 5‐year disease specific survival of 32–38% for grade 3 disease.^17^ The risk of progression may be as high as 88% by 2–3 years in high‐grade cases.^17^ In our cohort, we had a predicted 2‐year and 5‐year RFP of 74% (24, 92) and 63% (15, 85), respectively, if patients had undergone NU. The actual estimated 2‐year and 5‐year metastasis‐free probability in our cohort was comparable at 74% (53%, 100%) and 31% (7%, 100%). Understanding the limitations of staging and endoscopic management explains these poor outcomes and offers a rationale for MKP strategies. Patients with high‐grade disease may have microscopic invasive disease not detectable by imaging or the superficial‐depth biopsies obtained by ureteroscopy, nor confidently managed by superficial laser ablation, representing the source of progressive disease. Incorporating systemic therapy in these scenarios may provide a method to provide control for the unassessable invasive or micrometastatic component (Figure 2). These results, including the trifecta outcomes of 50% of patients maintaining kidney function and metastasis‐free status, provide a benchmark for future studies in these patients. It is important to balance the morbidity avoided by using MKP strategies with the oncologic risk of relapse and progression. Therefore, patients should be informed of the need to comply with stringent surveillance as recommended by the European Association of Urology.^2^ The decision to pursue MKP must be made on a case‐by‐case basis following a discussion with the patient.

In our series, all patients received systemic therapy as part of their definitive management, which was cisplatin‐based in only 29% (4 of 14) of patients. In the perioperative setting, there is paucity of data regarding the efficacy of non‐cisplatin‐based regimens in UTUC, and most of data are available from the adjuvant setting. The POUT study, which showed that adjuvant chemotherapy (compared with surveillance) significantly improved disease‐free survival (HR = 0.45, 95% CI 0.30–0.68), had allowed carboplatin‐based chemotherapy in 44% (55 of 126) patients.^10^ Subgroup analysis of disease‐free survival in carboplatin planned chemotherapy shows a univariable HR of 0.66 (95% CI 0.35–1.26) as compared with univariable HR of 0.35 (95% CI 0.20–0.61) in cisplatin‐planned chemotherapy, which suggests that clinical benefit was among cisplatin recipients. Our cohort of patients received a variety of non‐cisplatin‐based chemotherapy as a result of mainly renal contraindications (Table 3), as well as immunotherapy in 4 patients; furthermore, patients did not have their primary tumour resected, which contributes to the novel approach. Other factors beyond renal function were taken into consideration when systemic treatments were chosen; PS and neuropathy were also considered. Furthermore, in our cohort, suspicion of nodal disease on pretreatment cross‐sectional imaging and deficient mismatch repair lead to the choice of IA‐Gem and immunotherapy, respectively.

Limitations include the small sample size, the inherent selection bias associated with retrospective studies and lack of a comparator arm; use of the preoperative nomograms served as a way to provide a reference for expected disease outcomes if NU had been selected. Small sample size resulted in the imprecise estimates of 5‐year OS probability as compared with the 2‐year OS probability. The different endoscopic, topical and systemic therapies that patients received are also a limitation; however, they show that management of these patients is a case‐by‐case decision. The common features in our 14 patients were their need to avoid NU because of poor renal function and the predicted poor tolerability to dialysis and surgery. These patients appeared to have better survival compared with historical data with endoscopic‐only management and comparable outcomes to nomogram prediction, with the added benefit of renal preservation in a highly vulnerable situation. Given the lack of data with kidney preservation in high‐grade UTUC, prospective data with a larger cohort would be important to corroborate our observations and to inform clinical practice.

## 5 CONCLUSION

In patients with node‐negative localised or locally advanced high‐risk UTUC with relative contraindication to NU, management with MKP strategies was associated with good local and systemic control, was well tolerated and showed promising data on overall, recurrence, progression and dialysis‐free survival. These kidney‐preserving strategies require a multidisciplinary team approach and should be individualised for each patient after discussion of the benefits and the possibility of a non‐curative approach, as well as the need for close surveillance to avoid suboptimal oncologic outcomes.

## CONFLICT OF INTEREST

The authors declare no relevant conflict of interest pertinent to this study. Full conflict of inflict disclosures include research funding from BMS, Eisai, EMD Serono for A.Y.S; Honoraria from Pfizer, Roche, BMS and Exelixis for A.Y.S; and Urogen Pharma (consultant) and Urology Education (speaker) for S.F.M. SFM: grant from QED therapeutics; consulting fees Merck, J&J; honoraria Ology education, Clinical Care Options. NN: consulting Schlesinger Group; Ad Board Aduro Bio Tech; Stock Allogene Therapeutics. AMK: grants Adolor, BMS, FKD Industries, FerGene, Heat Biologics, Merck, Photocure, SWOG, NIH/GU SPORE, AIBCCR, Janssen (+ Taris), Seattle Genetics; consulting Arquer Diagnostics. Asieris, Biological Dynamics, Bristol Myers Squibb, CG Oncology, H3 Biomedicine/Eisai, Engene, FerGene, Imagin Medical, Janssen, Medac, Merck, Photocure, ProTara, Seattle Genetics, Sessen Bio, Theralase, US Biotest, Urogen Inc., Roche, TMC Innovation; patents CyPRIT (Cytokine Predictors of Response to Intravesical Therapy) Joint with UT MD Anderson Cancer Center; ad board: Arquer Diagnostics. Asieris, Biological Dynamics, Bristol Myers Squibb, CG Oncology, H3 Biomedicine/Eisai, Engene, FerGene, Imagin Medical, Janssen, Medac, Merck, Photocure, ProTara, Seattle Genetics, Sessen Bio, Theralase, US Biotest, Urogen Inc., Roche, TMC Innovation; leadership IBCG. MTC: grants or contracts ApricityHealth, Aravive, AstraZeneca, EMD Serono, Exelixis, Janssen, Pfizer; consulting Astellas, AXDev, AstraZeneca, Astellas, Exelixis, Eisai, EMD Serono, Exelixis, Genentech, Pfizer, SeaGen; honoraria Bristol Myers Squibb, Merck, Roche, Pfizer. AZ: grants or contracts Infinity pharma; consulting Amedo, Astra Zeneca, Bayer; honoraria Biocept, CancerNet, Incyte; other Janssen‐Cliag, McKesson Specialty Health, Pfizer. AOS: research grants or contracts: Basilea, Bristol Myers Squibb, Janssen, Merck Sharp and Dohme, Nektar Therapeutics; honoraria Janssen; Ad Board Astrazeneca, Basilea, Bavarian Nordic, Bristol‐Myers Squibb, Genentech, Ideaya Biosciences, Immunomedics, Janssen, LOXO‐Oncology, Merck sharp and Dohme, Mirati, Nektar Therapeutics, Seattle Genetics, Taiho.

## AUTHOR CONTRIBUTION

Conception and design of work: OA, SFM, AYS. Acquisition, analysis, and interpretation of data: OA, ACA, LX, SFM, AYS. Manuscript draft: OA, NRW. Revision of work and final approval of intellectual content: OA, MTC, LX, ACA, NRW, AOS, PGC, AZ, EJ, JG, MA, AMK, NN, LLP, CD, SFM, AYS.

## Supporting information


**Table S4.** Outcomes for 14 patients with localized upper tract urothelial carcinoma treated with multimodality kidney preserving strategies

**Table S5.** Outcome analysis.
