# Supplementary material for: Multimodal kidney‐preserving approach in localised and locally advanced high‐risk upper tract urothelial carcinoma
Source: BJUI Compass. 2021 Oct 11;3(1):37–44. doi: 10.1002/bco2.113 (PMC8988842; doi:10.1002/bco2.113)
Supplement: Supplementary file 1 — Table S4. Outcomes for 14 patients with localized upper tract urothelial carcinoma treated with multimodality kidney preserving strategies [file BCO2-3-37-s002.docx]

**Table 4. Outcomes for 14 patients with localized upper tract urothelial carcinoma treated with multimodality kidney preserving strategies**

| **Patient number** | **Age at diagnosis, years** | **Tumor location** | **Grade** | **ECOG PS** | **Baseline eGFR** | **Comorbidities** | **CCI, predicted 10-year survival** | **Prior urothelial cancer (management)** | **MMR** | **Chemotherapy** | **Immunotherapy** | **Best Radiographic Response** | **Progression site** | **Freifeld 5-yr RFP** | **Deceased** | **Survival (mo)** |
| --- | --- | --- | --- | --- | --- | --- | --- | --- | --- | --- | --- | --- | --- | --- | --- | --- |
| 1 | 72 | Ureter | High | 0 | 50 | n/a | 5, 21% | T1 bladder (BCG) | Proficient | IA-gem | None | Near CR | local | 0.5 | Yes | 48 |
| 2 | 77 | Renal pelvis | High | 2 | 76 | n/a | 6, 2% | Ta bladder (TUR)  LG ureter (TUR) | Proficient | Gem/cis | None | SD | Synchronus urothelial tract lesions | 0.65 | Yes | 77 |
| 3 | 73 | Renal pelvis | High | 1 | 87 | n/a | 5, 21% | T1 bladder (TUR)  LG ureter (TUR) | Proficient | CGI | None | CR | Synchronus urothelial tract lesions | 0.68 | Yes | 27 |
| 4 | 58 | Renal pelvis | High | 0 | 83 | n/a | 3, 77% | T2 distal ureter (ureterectomy alone) | Deficient | dd-MVAC | None | CR | None | 0.85 | No | 44 |
| 5 | 69 | Renal pelvis | High | 0 | 35 | Prior cisplatin  Prior nephrectomy | 4, 53% | T1 bladder and HG UC urter (BCG-refractory, ddMVAC🡪cystectomy+nephroureterectomy) | Unknown | None | Atezolizumab | NE | None | 0.63 | No | 26 |
| 6 | 78 | Renal pelvis | High | 0 | 40 | Prior nephrectomy, PVD, HTN | 7, 0% | HG Ureter (nephroureterectomy alone)  Ta bladder (BCG) | Unknown | None | Pembrolizumab | SD | Synchronus urothelial tract lesions | 0.62 | No | 36 |
| 7 | 78 | Renal pelvis | Low | 3 | 22 | Prior nephrectomy, HTN | 5, 21% | HG Ureter (nephroureterectomy alone)  Ta bladder (TUR) | Proficient | GTA | None | CR | metastatic disease | 0.31 | Yes | 24 |
| 8 | 59 | Renal pelvis | High | 1 | 52 | Prior carboplatin for rectal neuroendocrine tumor, prior nephrectomy | 4, 53% | HG Ureter (nephroureterectomy alone)  Ta bladder (TUR) | Proficient | Gem-cis | None | PR | Local recurrence | 0.84 | No | 31 |
| 9 | 80 | Renal pelvis | High | 2 | 30 | PVD, Chronic NSAIDs | 8, 0% | n/a | Proficient | Gem-carbo | Atezolizumab* | SD | Local recurrence | 0.63 | No | 19 |
| 10 | 89 | ureter | High | 1 | 43 | HTN | 6, 2% | n/a | Deficient | Gem-carbo | Nivolumab | PR | None | 0.2 | No | 44 |
| 11 | 76 | renal pelvis | High | 1 | 56 | DM2, HTN | 6, 2% | n/a | Deficient | None | pembrolizumab | SD | None | 0.65 | No | 7.5 |
| 12 | 63 | renal pelvis | High | 0 | 44 | prior nephrectomy, HTN | 4, 53% | HG Ureter (nephroureterectomy alone) | Deficient | None | Pembrolizumab | CR | None | 0.48 | No | 8 |
| 13 | 83 | ureter and renal pelvis | High | 1 | 38 | HTN, PVD, prior cisplatin | 7, 0% | HG ureter (Gem/Cis🡪ureterectomy) | Proficient | None | Pembrolizumab | CR | None | 0.1 | No | 14 |
| 14 | 71 | Renal pelvis | High | 0 | 25 | Prior nephrectomy for RCC, DM2 | 6, 2% | n/a | Proficient | GTA | none | PR | None | 0.6 | No | 22.5 |

*Pt 9 had poor tolerance to chemotherapy and was switched to atezolizumab

+Pt 10 was found to be microsatellite instability and was switched to nivolumab

ECOG PS: Eastern cooperative oncology group performance status, eGFR: estimated glomerular filtration rate, PVD: peripheral vascular disease, HTN: hypertension, NSAIDs: nonsteroidal anti-inflammatory drugs, DM2: diabetes mellitus type 2, RCC: renal cell carcinoma, CCI: Charlson Comorbidity Index, LG: low grade, HG: high grade, BCG: Bacillus Calmette Guerin, TUR: transurethral resection, MMR: mismatch repair. RFP: relapse-free probability. GTA: gemcitabine, taxotere, and adriamycin; CGI: cisplatin, gemcitabine, and ifosfamide; dd-MVAC: dose-dense methotrexate, vinblastine, adriamycin, and cisplatin; Gem/cis: gemcitabine and cisplatin; Gem/carbo: gemcitabine and carboplatin; IA-Gem: ifosfamide, adriamycin, and gemcitabine. CR: complete response, PR: partial response, SD: stable disease, NE: not evaluable.
