# Supplementary material for: Multimodal kidney‐preserving approach in localised and locally advanced high‐risk upper tract urothelial carcinoma
Source: BJUI Compass. 2021 Oct 11;3(1):37–44. doi: 10.1002/bco2.113 (PMC8988842; doi:10.1002/bco2.113)
Supplement: Supplementary file 2 — Table S5. Outcome analysis. [file BCO2-3-37-s001.docx]

**Table 5. Outcome analysis.**

| **Endpoint (N=14)** | **Median (95% CI) or frequency (%)** |
| --- | --- |
| **Overall survival (months)** | 48.1 (48.1, NA) |
| **5y OS rate** | 0.38 (0.09, 1) |
| **Progression-free survival (months)** | 22.4 (15.6, NA) |
| **Metastasis-free survival (months)** | 48.1 (26.8, NA) |
| **Dialysis-free survival (months)** | 48.1 (26.6, NA) |
| **Radiographic response:** | |
| **CR** | 5 (36%) |
| **PR** | 3 (21%) |
| **SD** | 3 (21%) |
| **NE** | 3 (21%) |
| **Required subsequent systemic therapy** | 9 (64%) |
| **Recurrence patterns:** | |
| **Local recurrence** | 7 (50%) |
| **Distant metastatic disease** | 3 (21%) |
| **Ultimate nephroureterectomy** | 3 (21%) |
| **Ultimate dialysis** | 2 (14%) |
| **Death during follow up period** | 4 (29%) |

M: Months, CI: confidence interval, NA: not applicable.
